# Personality, Preoperative Anxiety, and Postoperative Outcomes: A Review

**DOI:** 10.3390/ijerph191912162

**Published:** 2022-09-26

**Authors:** Wentao Ji, Chao Sang, Xiaoting Zhang, Keming Zhu, Lulong Bo

**Affiliations:** 1 Faculty of Anesthesiology, Changhai Hospital, Naval Medical University, Shanghai 200433, China; 2 Department of Anesthesiology, Shanghai General Hospital, Shanghai Jiao Tong University School of Medicine, Shanghai 201620, China

**Keywords:** personality, preoperative anxiety, postoperative outcome, anesthesia

## Abstract

Research has shown that personality is associated with anxiety levels in the general population. However, little is known about the relationship between personality and preoperative anxiety and the subsequent health outcomes in patients undergoing surgery. Therefore, this review aimed to identify studies that explored the relationship between personality traits and preoperative anxiety, as well as their association with postoperative outcomes. Existing literature shows that anxiety may play an intermediary role in the relationship between personality and postoperative outcomes. Severe anxiety may partially explain the adverse effects of certain personality traits, such as neuroticism, on postoperative outcomes. However, the relationship between personality traits, preoperative anxiety, and postoperative outcomes remains unclear. Interventions such as clinical evaluation, preoperative counseling, and management strategies can be of great value in identifying and resolving patients’ anxiety and negative emotions to improve postoperative outcomes.

## 1. Introduction

Personality refers to individual differences in characteristic and enduring patterns of behavior, feeling, and thinking. Each individual has their own personality traits. Although some studies have juxtaposed personality, along with anxiety and depression, as psychosocial factors, personality is a broader concept that may include mental or psychological traits [1,2,3]. Anxiety is conceptualized as a set of behavioral manifestations dominated by specific emotions, defined as a state in which there is an apprehensive or fearful anticipation of the future [4]. Depression and anxiety are common mental disorders in the general adult population, with prevalence rates of approximately 17% and 29%, respectively [5]. In the context of COVID-19, the incidence of anxiety and depression showed an increase due to pervasive uncertainty and fear of infection [6,7].

Anxiety is defined as a set of behavioral manifestations that can be divided into state and trait anxiety. State anxiety is a transitory emotional condition, while trait anxiety is a lifelong pattern of anxiety as a personality feature [8]. State anxiety is a subjective feeling experienced on encountering an anxiety-provoking stimulus, such as surgery; thus, preoperative anxiety is a kind of state anxiety [9,10]. Preoperative anxiety, generally considered a type of state anxiety related to impending surgery, is likely linked to individual personality traits and coping processes. A growing number of studies have indicated that preoperative anxiety is associated with increased and worse perioperative outcomes, including impaired wound healing, nausea, vomiting, and postoperative pain [11]. Furthermore, there is growing evidence that personality traits are associated with health status and prognostic outcomes in surgical patients, although the underlying mechanisms are unclear [12,13]. Thus, this study aimed to review existing studies to explore the subtle relationships between personality traits, preoperative anxiety, and postoperative health outcomes in patients undergoing surgery.

## 2. Methods

The studies included in this review were retrieved from various databases, including PubMed, EMBASE, and Cochrane Library, since their inception. A literature search was performed using the following keywords: personality, anxiety, preoperative, anesthesia, postoperative, outcome, and complications. No restrictions were applied on article type. The appropriateness of the inclusion criteria was determined by the authors to include a wide and unbiased range of relevant studies.

## 3. Personality, Health, and Patient Outcomes

Personality is the sum of all traits that distinguish an individual from others, encompassing the characteristic patterns of thoughts, feelings, and behaviors [1,2,3]. It is believed that personality arises from within an individual and remains fairly consistent throughout life. Several theories have been developed to understand human personality and personality traits, and various schools of thought in psychology have influenced many of these theories. Increasing research indicates that personality traits may impact individuals’ health, and that certain personality characteristics may be linked to illness [14]. Personality is also considered an important factor in the development of chronic diseases [15,16,17]. Many theories of personality type suggest that sometimes one personality type is more inclined to seek psychological resources to deal with stressful events, while another is more vulnerable and has a lower health-related quality of life [18].

The Big Five framework, also known as the five-factor model, is currently the most widely accepted personality theory. It states that personality can be divided into five core factors, known by the acronym CANOE or OCEAN: openness to experience, conscientiousness, extraversion, agreeableness, and neuroticism [19]. This theory of personality has been applied in many countries and cultural groups worldwide, and provides a reliable assessment scale for measuring personality [20]. In a meta-analysis of 11 studies with a total of 19,941 individuals, greater openness was associated with a lower risk of all-cause mortality after adjusting for standard mortality risk factors [21]. Similarly, Bunevicius et al. [12] reported that the personality trait of openness was associated with better cognitive function and a reduced risk of mortality in patients with benign brain tumors after surgery, independent of demographic and clinical risk factors including age, sex, and histological diagnosis. In a study exploring the relationship between diverse personalities and clinical outcomes in patients undergoing primary total knee arthroplasty, sanguine patients displayed the best clinical outcomes, while melancholic patients displayed the worst clinical outcomes [22]. These results suggest that perioperative personalized management based on a patient’s personality may show promise for recovery. A more recent study of 211 patients with total knee arthroplasty found that screening melancholic patients could significantly reduce in-hospital costs, avoid unnecessary suffering, and shorten the recovery period [23].

**Figure 1:**
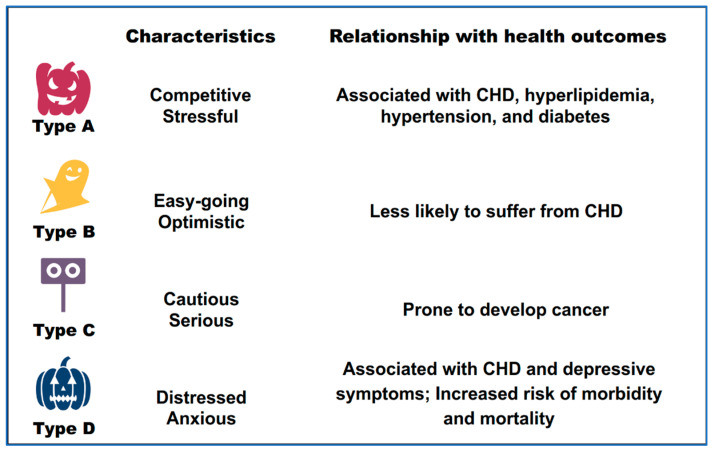
Personality types A, B, C, and D and their relationships with health outcomes. CHD: coronary heart disease.

Another theory, from the perspective of a close relationship with biological influences, divides personality into types A, B, C, and D (Figure 1). To be precise, this is an evolving theory as these four types of personalities are not comprehensive, that is, they do not define all types of personalities. Type A personality is characterized by competitiveness and is associated with coronary heart disease (CHD), high cholesterol, hypertension, diabetes, and stress [15]. Type B personality comprises traits such as easygoing, relaxed, and persuasive; people with this personality type are less likely to suffer from CHD. The behavior pattern of Type C personality is similar to that of Type A, but has a greater tendency toward perfectionism. Type C personality, also known as the cancer-prone personality, is characterized by a lack of negative feelings and a need for harmony [24]. Type D personality has two basic traits: negative affectivity and social inhibition. It is associated with impaired health conditions, such as CHD, increased depressive symptoms, increased risk of morbidity and mortality, and impaired quality of life [16].

The relationship between personality, cancer risk, and cancer prognosis has been extensively studied. Accumulating evidence indicates that Type C personality traits and neuroticism are associated with a higher risk of cancer and shorter survival period in patients with an established cancer diagnosis [25]. Potential underlying mechanisms linking personality traits with poor health status and worse prognosis of cancer include, but are not limited to, poor treatment adherence and adverse health behaviors [26]. Furthermore, personality traits can lead to the development of poor or adverse biological mechanisms that may predict tumor progression and worse prognosis [12,27].

Type D personality has been more widely studied and is closely related to the occurrence of various cardiovascular diseases and health outcomes of cardiovascular surgeries [13,28,29,30]. In a meta-analysis of over 5000 patients with CHD, a Type D personality was significantly associated with an increased risk of mortality and nonfatal myocardial infarction [28]. Denollet et al. [30] found that Type D personality was associated with long-term mortality and was a significant predictor of mortality in patients with CHD, after adjustment for other biomedical factors; furthermore, they noted that Type D personality was a predictor of prolonged acute brain dysfunction (delirium/coma) after cardiovascular surgery, and depressive symptoms associated with Type D personality traits increased the magnitude of acute brain dysfunction in these populations [13].

## 4. Personality and Preoperative Anxiety

Since the ancient Greeks, people have postulated a link between personality and mental health. The most famous example of an early theory is the doctrine of the four humors attributed to Hippocrates and Galen [31,32], which posited that personality types determine vulnerability to physical and mental illness. Within the general population, anxiety disorders are among the most common mental health illnesses worldwide, affecting approximately 29% of the population in their lifetime. Numerous empirical studies have examined the association between personality traits and anxiety disorders [5]. It is well established that the personality traits of individuals with anxiety disorders differ from those of individuals without anxiety disorders. According to a tripartite model of anxiety and depression, anxiety is characterized by elevated levels of negative affect and physiological hyperarousal [33]. Negative and positive affect are strongly linked to neuroticism and extraversion, respectively [34]. Hence, anxiety disorders are associated with high neuroticism and low extraversion [35,36]; conversely, personality traits such as high neuroticism and/or low extraversion may be a risk factor for certain anxiety disorders [37].

Interestingly, preoperative anxiety corresponded to state anxiety, whereas, personality traits such as neuroticism and type A personality corresponded to trait anxiety; furthermore, both state and trait anxiety were strongly correlated with each other, indicating that individuals with higher levels of trait anxiety experience increased state anxiety during anxiety-provoking situations such as surgery [8].

Undoubtedly, a strong connection exists between personality, anxiety, and health-related quality of life. Compared with the influence of personality on health, especially surgical outcomes, the effect of preoperative anxiety on the postoperative outcomes of surgical patients has been widely studied [38]. Before proceeding, it is necessary to clarify the relationship between personality and anxiety, especially preoperative anxiety, in the context of the current scientific literature. 

It is worth emphasizing, in particular, that anxiety and anxiety disorders are not the same. Anxiety disorders differ from developmentally normative fear or anxiety as they involve excessive anxiety that persists beyond developmentally appropriate periods and differs from transient fear or anxiety, often stress-induced, and is persistent (e.g., typically lasting 6 months or more), although the criterion for duration is intended as a general guide, allowing for some degree of flexibility [39]. Perioperative anxiety tends to be an anxious response to surgery. In one study, breast cancer patients with high preoperative anxiety levels also reported as persons with traits more prone to high levels of anxiety [40]. A study from Turkey showed that, among patients with breast cancer, those with high levels of extraversion had lower levels of anxiety and depression and maintained a better quality of life, whereas patients with higher neuroticism had higher levels of anxiety and an impaired quality of life [38]. Personality traits are also associated with emotional health in patients with brain tumors. Greater emotional stability, that is, lower neuroticism and greater openness, was associated with lower severity of depression and anxiety symptoms [12]. In contrast, lower openness and greater neuroticism were associated with depressive and anxiety disorders [41].

One reason why personality traits are associated with anxiety disorders may be that these traits and conditions are manifestations of common underlying genetic and/or environmental determinants [42,43,44]. In a sample of 3810 pairs of twins in Australia, Jardine et al. [45] found that genetic variations in anxiety symptoms heavily depend on the same factors that influence neuroticism. Similarly, Hettema et al. [46] found that the genes that influence neuroticism also influence the risk of generalized anxiety disorder. Although longitudinal studies of genetic information are needed to elucidate the role of heredity in the complex interaction between personality traits and anxiety, these studies have shown that personality and anxiety are closely linked at the genetic level. Family studies indicate that personality traits represent at least part of the heritability of anxiety disorders [47,48]. For example, trait anxiety and harm-avoidance were more common among relatives of patients with generalized social phobia, compared with relatives of patients who did not have this phobia [48,49].

The role of stress should not be neglected when clarifying the relationship between preoperative anxiety and personality traits. Stress may be defined as an external situation or stimulus, or as a physiological response, and its sources and effects on people are varied. Psychological stress refers to a relationship between the person and the environment, which is perceived by the person as exceeding his or her resources and threatening well-being [50]. Stress is a major contributor to anxiety, even in preoperative patients. Before surgery, stress arises from a fear of the unknown, including the risks associated with surgery and anesthesia, an unfamiliar medical environment, cost of therapy, possible pain, and even death, leading to preoperative anxiety [51,52]. People with different personality traits manage stress differently, and some can thrive in the same situations that cause severe anxiety for others. Research on personality has highlighted that constant and persistent personality traits can explain why some people are more vulnerable to stress than others [18]. In other words, personality may determine whether or not stress leads to anxiety. Research has also established associations between personality traits and anxiety, primarily neuroticism and extraversion, within the five-factor model or the Big Five [53,54,55]. Neuroticism is typically defined as a tendency toward anxiety, depression, self-doubt, and other negative feelings. Individuals with elevated levels of neuroticism respond poorly to environmental stress, interpret ordinary situations as threatening, and perceive minor frustrations as hopelessly overwhelming [56]. People with high neuroticism have been shown to experience more pronounced and less well-regulated responses to stressful life events [57,58]. Meanwhile, extraversion is often defined as a stable personality dimension, characterized primarily by the tendency to experience positive affect. It is commonly accepted that higher extraversion is potentially beneficial in coping with stress, as more extraverted persons tend to show lower reactivity to stressful situations, and are therefore less affected by stress [59]. However, the idea that extraversion confers benefits in coping with stress remains controversial and requires further investigation [60].

Similarly, individuals with Type A personality tend to be very competitive and self-critical, become easily stressed, and tend to overreact. With a constant sense of urgency, waiting before surgery can seem extraordinarily long and painful for patients with Type A personality [61], whereas individuals with Type B personality are more comfortable in stressful situations and experience lower levels of anxiety. Thus, the relationship between personality, stress, and anxiety clarifies that attitudes, strategies, or abilities to cope with stress form the basis of certain personality types. People with different personality types cope with stress in different ways, resulting in varying levels of anxiety.

## 5. Preoperative Anxiety and Postoperative Outcomes

Surgery is a huge source of various physical and mental stimuli for patients. Surgery may lead to anxiety, fear, pessimism, and other negative emotions in patients who may not have recovered from the emotional shock of the disease itself, and the ensuing anxiety is widely accepted as a normal response in preoperative patients [62,63]. Surgery-related stress will inevitably lead to negative emotions in some patients, which may affect postoperative outcomes. Previous studies have shown a strong association between anxiety and health outcomes in different patient populations [64,65,66,67]. Preoperative anxiety related to anesthesia remains a major concern for many patients [68]. Preoperative anxiety is also of great concern to anesthesiologists as it is a known risk factor for perioperative complications.

### 5.1. Epidemiology of Preoperative Anxiety

In accordance with anxiety found in the different patient populations described above, the incidence and degree of preoperative anxiety also varies [69]. Preoperative anxiety begins from treatment planning and gradually increases until it reaches its peak when entering the operating room [70]. The perioperative period is considered a stressful event, triggering specific emotional, cognitive, and physiological responses in patients awaiting surgery, and over two-thirds of patients report feeling anxious [71]. During the preoperative period, patients are exposed to various stressful situations that can lead to higher levels of stress [40]. A systematic review and meta-analysis of studies worldwide reported a pooled prevalence of 48% for preoperative anxiety among patients undergoing surgery [72]. However, the prevalence of preoperative anxiety varies according to the type of surgery, patients’ sex and age, and country [73].

The type of surgery is an important factor affecting the prevalence of preoperative anxiety. Studies conducted in Europe have shown that the prevalence of preoperative anxiety among patients undergoing surgery varies from 27% to 80%. Hellstadius et al. reported that 34% of esophageal cancer patients experienced pre-surgical anxiety; 15% of them had mild anxiety, 14% had moderate anxiety, and 5% had severe anxiety [74]. Preoperative anxiety in patients undergoing cardiac surgery is much more common, as the risks associated with such complex operations make patients feel insecure. This is evidenced by the findings of Prado-Olivares et al., who showed that anxiety was identified in 80% of patients, and 40% of patients had high anxiety before surgery [75]. 

Age is believed to be negatively correlated with preoperative anxiety; patients aged below 50 years had a significantly higher incidence and degree of preoperative anxiety than those aged more than 50 [76,77]. However, some studies have shown that older patients experience higher levels of preoperative anxiety than younger patients because of comorbidities [78]. Research has also shown that age is not a risk factor for preoperative anxiety [79]. Next, sex was found to be an influential factor and a predictor that had a positive significant correlation with preoperative anxiety, with female sex being associated with higher levels of anxiety [77,80,81,82]. Among female patients, preoperative anxiety before elective cesarean section was more extensive and severe, with an incidence of approximately 72.7% [73]. The reasons for this could be physical discomfort and concerns about the safety of the fetus.

The prevalence of preoperative anxiety varies by country and region. Among patients undergoing total knee arthroplasty, the incidence of preoperative anxiety was 20.2% in the United States [83] and 22.7% in the Netherlands [84]. In line with previous findings that preoperative psychological problems appear to be more serious in Asians [85], the incidence of preoperative anxiety prior to total knee arthroplasty was 45.24% in Chinese patients [86]. These differences may be due to a variety of factors, such as socioeconomic characteristics and culture, which are also key environmental factors that contribute to the different expression of personality traits.

The type of anesthesia may also influence the occurrence of preoperative anxiety. Patients who were subjected to spinal anesthesia had a significantly lower incidence and severity of preoperative anxiety than those under general anesthesia [70,87]. Maheshwari et al. reported that the prevalence of preoperative anxiety was significantly higher in patients who received general anesthesia (97.18%) compared with those who received regional anesthesia (51.81%) for elective cesarean section [73].

In addition, emergency surgery, education level, family support, and previous surgical experience may also affect preoperative anxiety [70,73,80,88]. However, the influence of these factors on preoperative anxiety is complex, and further research is needed to identify and clarify these associations. Currently, the widely accepted view is that patients with higher preoperative anxiety are younger, female, undergoing major or emergency surgery under general anesthesia, and have family support.

### 5.2. The Influence of Preoperative Anxiety on Postoperative Outcomes

Most patients are anxious during the preoperative period as a natural reaction to unpredictable and potentially threatening situations [89,90]. It has been shown that a curved, rather than a simple linear relationship, exists between preoperative anxiety and postoperative complications; for instance, the incidence of postoperative complications was significantly lower in patients with mild anxiety than in those without anxiety or with moderate-to-severe anxiety [91,92,93]. Although such a conclusion is still controversial [94], it is possible that mild anxiety promotes adaptive actions to cope with jeopardizing stimuli, thereby improving postoperative outcomes [91,93]. However, it has been proved that excessive anxiety negatively affects patients’ outcomes, as demonstrated among patients in a variety of medical settings [9,64,65,95,96,97]. Excessive degrees of preoperative anxiety induced by real or even implied threats can activate the stress axis, namely, the hypothalamic-pituitary-adrenocortical (HPA) system, which in turn induces multisystem physiological responses, primarily in the cardiovascular system [98,99]. These responses include tachycardia, hypertension, arrhythmias, and higher levels of pain, which may persist in the postoperative period [100]. The impact can be multidimensional and dramatic, with serious physical and mental consequences [101]. The adverse effects of preoperative anxiety on perioperative outcomes of patients are roughly divided into four aspects related to patients’ feelings, medical intervention, complications, and postoperative recovery.

Pain is a very common, complex, subjective, and emotional sensory experience with both physiochemical and affective components. Pain can be a source of anxiety that increases its incidence and severity. Preoperative anxiety has long been recognized as a significant predictor of postoperative pain [96,102]. Studies have reported that preoperative state anxiety was positively correlated with postoperative pain [9,96,97]; the underlying mechanisms may be that anxiety is associated with a lower pain threshold [66], overestimation of pain intensity [103], and activation of the entorhinal cortex of the hippocampal formation [104]. The level of preoperative anxiety is closely related to postoperative pain, discomfort, satisfaction with recovery from surgery, and psychosocial outcomes; high levels of anxiety are associated with higher levels of discomfort and dissatisfaction [105,106]. 

Van Den Bosch et al. showed an increased incidence of postoperative nausea and vomiting depending on the level of preoperative anxiety [64]. Furthermore, perioperative cardiac events may lead to irreversible consequences, and untreated anxiety is associated with major cardiac events in cardiac patients [65], such as congestive heart failure, acute myocardial infarction, and pulmonary edema. A study from Korea found that the State-Trait Anxiety Inventory (STAI) score was useful for predicting hemodynamic responses during anesthesia induction in non-cardiac surgical patients [10] Moreover, levels of patient-reported preoperative anxiety independently predicted the risk of mortality and major morbidity in patients aged >70 years undergoing cardiac surgery [95]. A recent systematic review and meta-analysis, including 16 studies and 236,595 patients undergoing cardiac surgery, revealed that perioperative anxiety is associated with increased postoperative mortality [65].

Preoperative anxiety can contribute to resistance to anesthetics, thereby increasing the dosage of anesthetic needed intraoperatively, which makes patients more vulnerable to unfavorable events, including delayed recovery from anesthesia [67,107]. It has been shown that patients with higher anxiety scores (both state and trait anxiety) required greater amounts of propofol to attain light and moderate levels of sedation [9]. Similarly, high levels of preoperative anxiety can lead to increased postoperative analgesic use. Assessing preoperative anxiety levels could theoretically help guide perioperative anesthetic and analgesic doses. The association between increased levels of preoperative anxiety and prolonged hospital stays and frequent readmission is also well established [11,67,108]. Furthermore, excessive anxiety triggers a physiological stress response that can impede wound healing [109]. Studies have shown that preoperative anxiety also plays a role in increasing the risk of infection and weakening the immune response [110]. In patients with severe preoperative anxiety, the ability to return to daily activities and pre-surgery quality of life may be affected [9]. Thus, preoperative anxiety has a negative impact on postoperative outcomes in patients [111].

### 5.3. Strategies to Mitigate Preoperative Anxiety and Improve Postoperative Outcomes

Preoperative anxiety, even at high levels, does not generally meet the clinical diagnosis of general anxiety disorders. Importantly, preoperative anxiety is potentially modifiable, and identifying these patients may provide an opportunity to increase psychological comfort, thereby improving postoperative outcomes [95].

Anxiety assessment scales are commonly used to assess and identify preoperative anxiety. Currently, the five most widely used anxiety scales in English are the Arthritis Impact Measurement Scales, Hospital Anxiety and Depression Scale (HADS), Beck Anxiety Index, Zung Anxiety Inventory, and State-Trait Anxiety Inventory (STAI) [112]. Given the high rates of previously undiagnosed psychological conditions, preoperative psychological assessment is now a prerequisite for providing timely and appropriate interventions in some medical settings [113]. The administration of a simple screening questionnaire may warrant further studies for rapid bedside evaluations. A quick evaluation of anxiety symptoms as part of the preoperative visit may allow the identification of high-risk patients, and subsequently, appropriate pharmacological or psychotherapeutic interventions may be applied [40,95].

Current strategies for managing preoperative anxiety include both medical and nonmedical interventions [107]. Anesthesiologists may prefer medical interventions, such as benzodiazepines, while nonmedical interventions require collaboration between anesthesiologists, nurses, and surgeons. Many nonmedical strategies to reduce patient anxiety have been attempted; among these, music therapy and perioperative patient education have proven to be effective interventions [114]. Communication therapy, including preoperative education, is helpful in alleviating fear and stress [90]. Long wait times before surgery, with little information, add to the degree of anxiety. Anxious patients felt that their concerns were not fully or clearly responded to and complained frequently about insufficient information, inadequate respect, and insufficient empathy [115]. These factors increase anxiety among patients and their family members. Good communication and extensive preoperative counseling can alleviate mental stress and reduce moderate to severe anxiety in patients [91,116]. The anesthesiologist’s visit, the surgeon’s interview, and the nurse’s attention are all indispensable for doctor-patient communication before surgical procedures, providing multiple opportunities to dispel doubts and moderate anxiety. A previous study indicated that providing an audiotaped recording of the consultation before cardiac surgery improved patients’ knowledge and perceptions of control over their health status, thereby reducing anxiety and depression [117]. However, a review of randomized controlled trials evaluating the effects of preoperative education on anxiety and clinical outcomes in cardiac surgical patients showed inconsistent results [118]. The findings suggested that preoperative communication is not the same as simply informing patients of the risks of surgery or anesthesia, nor is it comprehensive and detailed medical knowledge. It is conceivable that some individuals do not want as much information as others; thus, a framework of general information should be established for individuals, and more opportunities should be provided for patients in terms of focusing on their real needs [114]. Effective and sound preoperative communication provided by medical staff requires patience, empathy, and skills, and should be implemented based on individual cultural background, language, and religious preferences [119].

Music therapy is also a simple and accessible intervention with proven effectiveness in reducing preoperative anxiety [120,121]. A previous study found that exposure to music significantly reduced self-reported anxiety preoperatively, intraoperatively, and postoperatively, and improved patients’ experience of cataract surgery [122]. Listening to music can reduce sympathetic nervous activity, allowing patients to relax emotionally and physically [123]. Playing pleasant music on headphones can also mask annoying noises existing in medical settings and distract patients from the anxiety induced by auditory stimuli [124]. In addition, exposure to music has been found to significantly reduce analgesic and sedative consumption [125], relieve postoperative pain [124] and improve patient satisfaction [126], which may be associated with reduced anxiety. Interestingly, the genres and types of music also need to be considered. A clinical trial concluded that classical Western music was more effective in reducing anxiety during dental surgery, compared with Turkish music and soft rock music [127]. As the popularity of mobile phones has made listening to music more convenient than ever, this strategy is worthy of further research. Although research has shown the benefits of personalizing one’s favorite music, professional psychologists should make appropriate recommendations regarding the type of music and a single cycle versus switching tracks for music therapy. In addition, other strategies such as using essential oils, watching television, and using relaxation techniques have been considered as alternatives to relieve anxiety, but little reliable evidence is currently available for these [107,114].

## 6. Discussion

**Figure 2:**
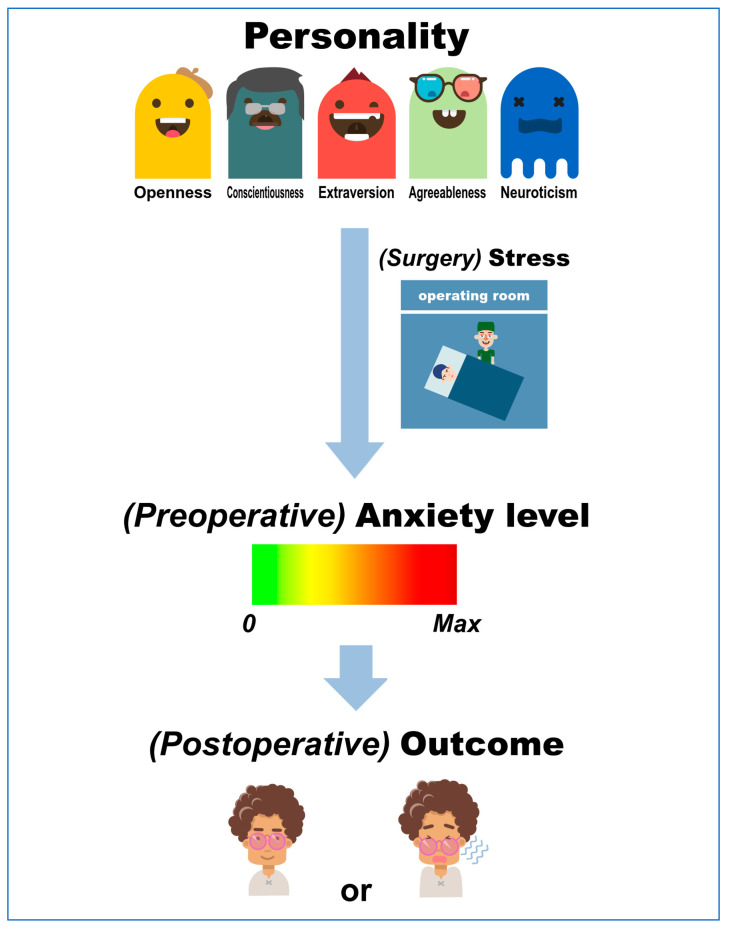
The mechanism underlying the relationships between personality traits, preoperative anxiety, and postoperative outcomes. The Big Five framework was used to describe personality.

Personality traits and levels of preoperative anxiety vary from one individual to another, which has complex and distinct impacts on patient outcomes. Furthermore, individuals with different personalities possess varying abilities to cope with stress, which plays a role in postoperative outcomes. This review focused mainly on the Big Five model of personality, preoperative anxiety, and postoperative outcomes and aimed to provide an updated overview of the current literature (Figure 2).

Many studies have focused on preoperative anxiety and reported relevant management strategies that can effectively reduce the incidence and severity of preoperative anxiety, thereby improving postoperative outcomes. Compared with preoperative anxiety, personality is a constant feature shaped by multiple factors over a long period and is not easily changed. As a complex psychological reaction, anxiety is influenced by a variety of factors related to personal experiences, including specific sociodemographic and personality factors [128]. Therefore, appropriate preoperative education and counseling should be offered based on each patient’s cultural background, personality traits, language, and religious preferences [119]. To develop appropriate interventions for the specific psychological needs of patients, further research is needed to evaluate preoperative anxiety screening procedures and clarify the nature and timing of support, which may require the involvement of psychological experts.

Although personality is associated with the occurrence of certain diseases and health outcomes, few studies have examined the association between personality and postoperative outcomes in patients undergoing surgery [13,22]. In contrast, the relationship between personality and anxiety, as well as that between preoperative anxiety and postoperative outcomes, is much clearer. Based on current findings, anxiety seems to be a major factor in the relationship between personality and postoperative outcomes; however, further evidence is needed to determine the effect of personality on the outcomes of patients undergoing surgery.

Our review should be interpreted in light of several limitations. Pertaining to personality with a variety of classification models, this diversity increases the complexity of exploring the relationship between personality and postoperative outcomes. This review almost exclusively focused on the Big Five personality model, mainly because this model is widely accepted and used in clinical research. However, focusing on the Big Five model may have led to ignoring the correlations with other personality dimensions; for example, people with harm-avoidance personality, as per the Temperament and Character Inventory, may be a high-risk population for preoperative anxiety [49]. However, the purpose of this review was to provide justification for the hypothesis that personality is related to postoperative outcomes, rather than to define an explicit relationship between the two, which compensate for not being able to fully explore complex personality models. Although we performed a comprehensive literature search within several databases, we may have missed relevant literature in other databases and languages. Furthermore, we did not perform a systematic or scoping review to determine the effect of personality on preoperative anxiety, and then the postoperative outcomes. Therefore, the strategies for managing preoperative anxiety are not discussed in great detail, and deserve further investigation. In addition, the current review primarily focused on the adult population, rather than children. Thus, the conclusion may not be applicable to other populations. Future systematic reviews should be conducted to provide corresponding interventions for existing or underlying preoperative anxiety.

## 7. Conclusions

In conclusion, anxiety plays an intermediary role in the relationship between personality traits and postoperative outcomes. Severe anxiety may partially explain the adverse effects of certain personality traits, such as neuroticism, on postoperative outcomes. However, the relationship between personality traits, preoperative anxiety, and postoperative outcomes remains unclear. Studies exploring whether personality is correlated with postoperative outcomes in surgical patients, both before and after preoperative anxiety management, are also worthwhile. In addition, interventions such as clinical evaluation, preoperative counseling, and management strategies can be of great value in identifying and resolving anxiety and negative emotions to improve postoperative outcomes, while the patients with clinically significant psychological conditions should be referred for psychological intervention.

## Data Availability

Not applicable.
